# Supplementary material for: Raman Study of 532-Nanometer Laser-Induced Degradation of Red Lead
Source: Materials (Basel). 2024 Feb 6;17(4):770. doi: 10.3390/ma17040770 (PMC10890575; doi:10.3390/ma17040770)
Supplement: Supplementary file 1 [file materials-17-00770-s001.zip › materials-2801669-supplementary.pdf]

Raman Study of 532-Nanometer Laser-Induced Degradation of Red Lead

Yan Li, Junjie Ma, Kang He, and Fengping Wang *

Department of Physics, School of Mathematics and Physics, University of Science and Technology Beijing, Beijing 100083, China; liyan000998@163.com (Y.L.); b20150328@xs.ustb.edu.cn (K.H.)

* Correspondence: fpwang@ustb.edu.cn

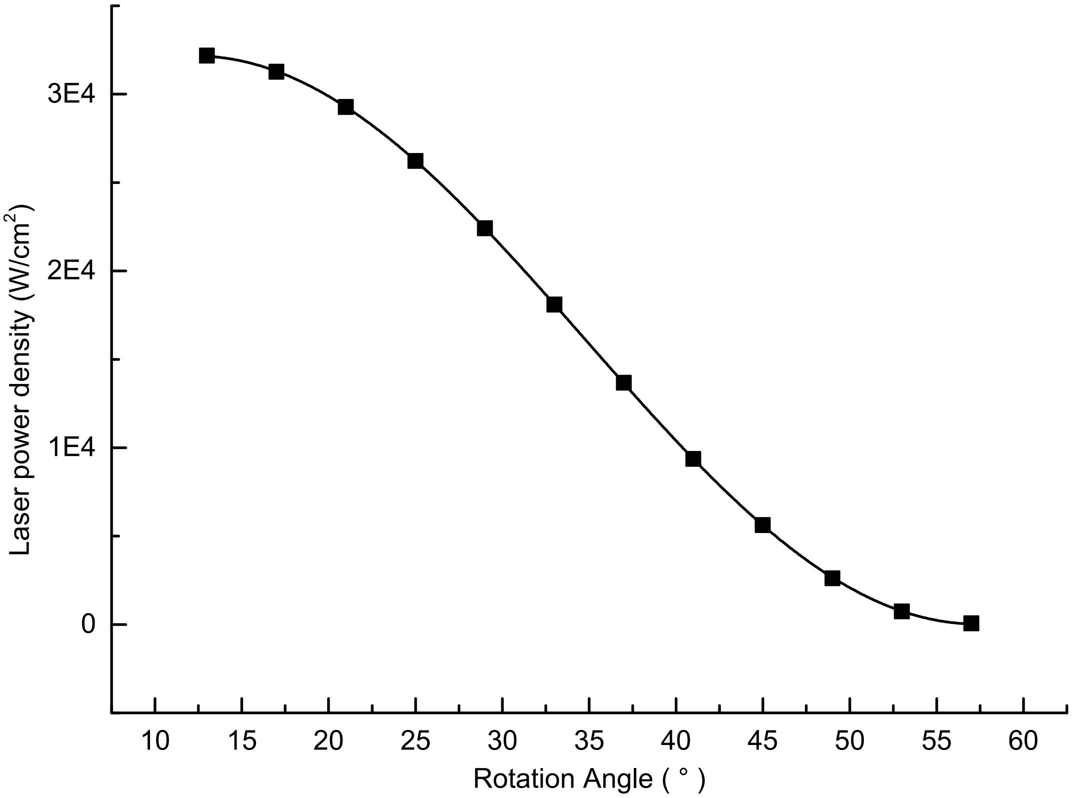

Figure S1 The laser power density of 532 nm (50 X objective) at different rotation angles of the control system

Table S1 The calculated temperature of red lead by using the ratio of the Stokes and anti-Stokes intensity.

Temperature/℃	Calculated temperature/℃	Error/℃
25	27	2
110	135	25
210	208	2
310	371	61
410	145	265
510	117	393
610	462	148
